# Molecular Mechanisms Underlying the Biosynthesis of Melatonin and Its Isomer in Mulberry

**DOI:** 10.3389/fpls.2021.708752

**Published:** 2021-10-06

**Authors:** Sha Zheng, Yingxue Zhu, Changying Liu, Shuai Zhang, Maode Yu, Zhonghuai Xiang, Wei Fan, Shuchang Wang, Aichun Zhao

**Affiliations:** ^1^State Key Laboratory of Silkworm Genome Biology, Key Laboratory of Sericultural Biology and Genetic Breeding, Ministry of Agriculture, Southwest University, Chongqing, China; ^2^Shaanxi Academy of Traditional Chinese Medicine, Xi’an, China; ^3^Key Laboratory of Coarse Cereal Processing, Ministry of Agriculture and Rural Affairs, Chengdu University, Chengdu, China; ^4^Institute of Environment and Plant Protection, Chinese Academy of Tropical Agricultural Sciences, Haikou, China

**Keywords:** mulberry, melatonin, melatonin isomers, biosynthesis, *N*-acetylserotonin methyltransferase (ASMT)

## Abstract

Mulberry (*Morus alba* L.) leaves and fruit are traditional Chinese medicinal materials with anti-inflammatory, immune regulatory, antiviral and anti-diabetic properties. Melatonin performs important roles in the regulation of circadian rhythms and immune activities. We detected, identified and quantitatively analyzed the melatonin contents in leaves and mature fruit from different mulberry varieties. Melatonin and three novel isoforms were found in the *Morus* plants. Therefore, we conducted an expression analysis of melatonin and its isomer biosynthetic genes and *in vitro* enzymatic synthesis of melatonin and its isomer to clarify their biosynthetic pathway in mulberry leaves. *MaASMT4* and *MaASMT20*, belonging to class II of the *ASMT* gene family, were expressed selectively in mulberry leaves, and two recombinant proteins that they expressed catalyzed the conversion of *N*-acetylserotonin to melatonin and one of three isomers *in vitro*. Unlike the *ASMT*s of *Arabidopsis* and rice, members of the three *ASMT* gene families in mulberry can catalyze the conversion of *N*-acetylserotonin to melatonin. This study provides new insights into the molecular mechanisms underlying melatonin and its isomers biosynthesis and expands our knowledge of melatonin isomer biosynthesis.

## Introduction

Melatonin, a kind of biogenic indolamine, is a pineal secretory product and was first discovered in the bovine pineal gland in 1958 (Lerner et al., 1958). Since then, melatonin has been detected in other species, such as bacteria, algae, fungi, animals, and plants (Pöggeler et al., 1991; Hattori et al., 1995; Manchester et al., 1995; Sprenger et al., 1999). Melatonin performs important roles in the regulation of many physiological processes, such as circadian rhythms, sleep, mood, body temperature, appetite, retina physiology and immune system (Lieberman et al., 1984; Lewy et al., 1992; Dollins et al., 1994). Additionally, melatonin is a free radical scavenger and acts as a wide-spectrum antioxidant to scavenge hydroxyl radicals and hydrogen peroxide (Reiter, 1998; Stasica et al., 1998; Reiter et al., 2013).

Melatonin was first reported in plants in 1995, and is contained in almost all tested plants, at concentrations ranging from pico- to nano-grams per gram of tissue (Garcia-Parrilla et al., 2009; Gomez et al., 2013). Although differing melatonin concentrations have been reported in some plants and fermentation products, there is limited information on melatonin isomers (Rodrigueznaranjo et al., 2011; Kocadagli et al., 2014). Melatonin isomers were first reported in wine using HPLC coupled with mass/mass spectrometry (MS/MS), and the presence of melatonin isomers has been confirmed (Rodrigueznaranjo et al., 2011; Gomez et al., 2012, 2013; Vitalini et al., 2013). The distributions of melatonin and its isomers vary widely in wine products (Gomez et al., 2013). Melatonin isomers have been identified in other fermentation and plant products, such as bread, beer, and orange juice (Fernández-Pachón et al., 2013; Kocadagli et al., 2014). Recently, a melatonin isomer was found in sesame extract (Vitalini et al., 2020). Based on the data of Diamantini et al. (1998), isomer structures in wine were tentatively identified as 1-(2-alkanamidoethyl)-6-methoxyindole and *N*-acetyl-3-(2-aminoethyl)-6-methoxyindole (Tan et al., 2012).

The melatonin biosynthetic pathway was characterized earlier in animals. The source of melatonin is L-tryptophan, and it is produced when tryptophan hydroxylase (TPH) catalyzes the production of 5-hydroxytryptophan by tryptophan (Fitzpatrick, 1999). Then, 5-hydroxytryptophan is catalyzed by aromatic amino acid decarboxylase (AADC) to serotonin, acetyltransferase catalyzes the formation of serotonin to *N*-acetyl-5-hydroxytryptamine, and finally *N*-acetyl-5-hydroxytryptamine is catalyzed by *O*-methyltransferase (OMT) to melatonin (Arnao and Hernández-Ruiz, 2014; Huang et al., 2017). The melatonin synthetic pathways in plants and animals differ. For example, in plants, in the initial step, tryptophan is decarboxylated to form tryptamine instead of being hydroxylated to 5-hydroxytryptophan, which is then hydroxylated to form serotonin (Back et al., 2016). Thereafter, the other melatonin biosynthetic processes are similar to those in animals. These steps involve serotonin *N*-acetyltransferase (SNAT) and *N*-acetylserotonin methyltransferase (ASMT) (Kang et al., 2011, 2013). However, the biosynthesis of melatonin isomers is still completely unclear (Gomez et al., 2013).

Mulberry (*Morus alba* L.) is an important medicinal herb with multiple functions, such as antioxidant, anti-cancer and hypoglycemic, and is involved in regulating immunity and sleep (Chen et al., 2003; He et al., 2013; Sánchez-Salcedo et al., 2015). According to the literature, mulberry leaves and fruit contained higher melatonin content than other tissues (Chen et al., 2003; Pothinuch and Tongchitpakdee, 2011; Wang et al., 2016). In addition, the melatonin contents among different varieties of the same species of plant could be significantly different by several 100-fold (Vitalini et al., 2013; Wang et al., 2016). We detected, identified and quantitatively analyzed the melatonin contents in leaves and mature fruit of different mulberry varieties. We found that melatonin isomers were present from all the tested mulberry varieties. The complete sequence of the mulberry genome is available and provides an opportunity for the characterization of biosynthetic genes involved in melatonin and its isomers (He et al., 2013; Jiao et al., 2020). Thus, we identified 37 putative melatonin and its isomer biosynthetic genes and analyzed their expression patterns and enzyme functions to clarify the biosynthetic pathway of melatonin and its isomer in mulberry (Zheng et al., 2021). The results provided insights into the molecular mechanism underlying melatonin and its isomer biosynthesis in mulberry and expanded our knowledge of melatonin isomer biosynthesis.

## Materials and Methods

### Plant Materials

The leaves were collected from different varieties of mulberry trees. All the mulberry trees were grown without chemical pesticides, and without of wild silkworms (Table 1 and Supplementary Table 1). Mulberry (*Morus* spp.) plants were grown at the Mulberry Germplasm Nursery in Southwest University, China. The mature mulberry leaves of 50 mulberry varieties were harvested in May, 2015. The mature mulberry leaves were harvested at 10: 00 AM on April 28th, June 28th, and August 28th, 2016. Leaves at different maturation stages (represented by the 1st, 5th, 10th, 15th, and 20th leaf positions) from three varieties were harvested per variety in July, 2016. The 1st leaves were selected from positions of 1∼3 from the top of each branch, the 5th leaves were from positions 4 and 5, the 10th leaves were from positions 9 and 10, the 15th leaves were from positions 14 and 15, and the 20th leaves were from positions 19 and 20. These mulberry leaves were oven-dried at 50°C to a constant mass and then pulverized. The powders were passed through a 100-mesh sieve and stored at −40°C until used.

**TABLE 1:** Contents of melatonin and its isomers in the mature mulberry leaves from 50 varieties harvested in May, 2015.

**Name of mulberry variety**	**Specie name of mulberry variety**	Name of sample*	**Mel (ng/g) (DW)**	**SD**	**MI-1 (ng/g) (DW)**	**SD**	**SUM (ng/g) (DW)**
Naxi	*Morus sp.*	S1-L	nd	0	0.23	0.03	0.23
Chuansang	*Morus notabilis* C. K.	S2-L	0.83	0.01	0	0.01	0.83
Zhichuisang	*Morus multicaulis* Perr.	S3-L	nd	0	0.99	0.08	0.99
Jialing-NO.30	*Morus multicaulis* Perr.	S4-L	0.36	0.02	0.63	0.06	1
Zhenzhubai (2X)	*Morus alba* Linn	S5-L	nd	0	1.03	0.04	1.03
Hongguo-NO.2	*Morus atropurpurea* Roxb.	S6-L	nd	0	1.2	0.13	1.2
Jialing-NO.40	*Morus multicaulis* Perr.	S7-L	0.37	0.05	0.85	0.08	1.22
Jialing-NO.20	*Morus multicaulis* Perr.	S8-L	0.43	0.01	0.82	0.16	1.26
Xiaoguansang	*Morus multicaulis* Perr.	S9-L	nd	0	1.27	0.05	1.27
Zhongsang5801	*Morus multicaulis* Perr	S10-L	nd	0	1.36	0.09	1.36
Zhenzhubai (4X)	*Morus alba* Linn.	S11-L	nd	0	1.44	0.15	1.44
Dahuayepisang	*Morus cathayana* Hemsl.	S12-L	nd	0	1.66	0.06	1.66
Hongguo-NO.1	*Morus multicaulis* Perr.	S13-L	nd	0	1.75	0.05	1.75
Sri Lanka2	*Morus bombycis* koidz.	S14-L	nd	0	1.77	0.1	1.77
Yuanyepisang	*Morus alba* Linn.	S15-L	nd	0	1.9	0.05	1.9
Guofeng	*Morus sp.*	S16-L	nd	0	2.01	1	2.01
Sri Lanka1	*Morus bombycis* koidz.	S17-L	nd	0	2.56	0.21	2.56
Jianchi	*Morus bombycis* koidz.	S18-L	nd	0	2.62	0.69	2.62
Yanbian	*Morus alba* Linn.	S19-L	nd	0	2.68	0.34	2.68
Georgia mulberry	*Morus multicaulis* Perr.	S20-L	nd	0	2.76	0.23	2.76
Xinyizhilai (2X)	*Morus alba* Linn.	S21-L	nd	0	2.97	0.39	2.97
GM31	*Morus sp.*	S22-L	nd	0	3.11	0.05	3.11
Hongyousang	*Morus alba* Linn.	S23-L	nd	0	3.58	0.02	3.58
Ya’an-NO.7	*Morus sp.*	S24-L	nd	0	4.06	0.32	4.06
Baiyuwang	*Morus multicaulis* Perr.	S25-L	nd	0	4.09	0.4	4.09
Xinong6071	*Morus bombycis* koidz.	S26-L	0.58	0.02	3.51	0.21	4.09
Yidachimu	*Morus bombycis* koidz.	S27-L	nd	0	4.55	0.09	4.55
Xiqing-NO.3	*Morus bombycis* koidz.	S28-L	nd	0	5.15	0.18	5.15
Ya’an-NO.1	*Morus sp.*	S29-L	nd	0	5.81	0.42	5.81
Cambodia mulberry	*Morus sp.*	S30-L	nd	0	5.93	0.18	5.93
Xiqing-NO.5	*Morus multicaulis* Perr.	S31-L	nd	0	6.13	0.52	6.13
Qiangbing	*Morus sp.*	S32-L	nd	0	6.18	0.28	6.18
Jialing-NO.16	*Morus alba* Linn.	S33-L	0.46	0.01	5.89	0.25	6.35
Laos mulberry	*Morus sp.*	S34-L	nd	0	6.79	0.2	6.79
Shan305	*Morus alba* Linn.	S35-L	nd	0	7.09	0.57	7.09
Huosang-NO.1	*Morus mizuho* Hotta.	S36-L	nd	0	7.26	0.33	7.26
Yunnanchangguosang	*Morus laevigata* Wall.	S37-L	nd	0	7.96	0.33	7.96
Guangyuan-NO.5	*Morus sp.*	S38-L	nd	0	8.22	0.79	8.22
Tongxiangqing	*Morus multicaulis* Perr.	S39-L	nd	0	8.45	1.01	8.45
Xiqing-NO.1	*Morus alba* Linn.	S40-L	nd	0	8.65	0.54	8.65
Zijin	*Morus alba* Linn.	S41-L	nd	0	8.8	0.63	8.8
Xinongxinyizhilai (4X)	*Morus alba* Linn.	S42-L	nd	0	9.09	0.14	9.09
Lunjiao40	*Morus atropurpurea* Roxb.	S43-L	0.26	0.02	9.84	0.44	10.1
Xiqing-NO.4	*Morus multicaulis* Perr.	S44-L	nd	0	10.28	0.55	10.28
Xiqing-NO.2	*Morus bombycis koidz.*	S45-L	nd	0	10.3	1.79	10.3
Ganluo-NO.6	*Morus sp.*	S46-L	nd	0	10.64	0.12	10.64
India mulberry	*Morus indica L.*	S47-L	nd	0	12.67	0.58	12.67
Xiaohuayepisang	*Morus cathayana* Hemsl.	S48-L	nd	0	14.39	0.26	14.39
Shenlongjiachangsuisang	*Morus wittiorum Hand.*-Mazz.	S49-L	0.51	0.01	18.99	0.17	19.5
Cesha	*Morus alba* Linn.	S50-L	nd	0	20.58	0.76	20.58
	Average						5.49 ng/g

*The nomenclature of Name of sample is S for Sample, Arabic numerals for the number of mulberry variety, and the last capital letter for tissue of mulberry. For example, S1-L is S for Sample, 1 for the number of mulberry variety “Naxi,” and L for leaves of mulberry variety “Naxi.” “Mel” is melatonin. “MI-1” is melatonin isomer-1. “nd” is no detected. “sp.” indicate that no species name has been determined. Data are means ± SDs (n = 3).

Mulberry varieties “Dashi,” “Baiyuhuang,” “Jialing NO. 30,” and “Zhongsang 5801” are the same materials used to detect melatonin and isomers and to clarify the molecular mechanisms of their biosynthesis. These mulberry varieties grown in the mulberry field of the Chongqing Sericulture Science and Technology Research Institute, China and were harvested in April 2017 (Supplementary Table 1). Mulberry varieties “Dashi,” “Baiyuhuang,” “Jialing NO. 30,” and “Zhongsang 5801” grown in natural conditions. Parts of the mature mulberry leaves and fruit were picked, lyophilized and ground into powders. The fruits were collected at 33–37 days after full-bloom (Liu et al., 2015). They were then stored in a −40°C refrigerator for the detection of melatonin and its isomers. Other parts of the samples were immediately frozen in liquid nitrogen and stored at −80°C for molecular biology experiments.

### Reagent and Chemicals

All the experimental event sequences were controlled using UNIFI software (Waters). Melatonin (CAS NO.73-31-4), *N*-acetylserotonin (CAS NO.1210-83-9) and tryptophan ethyl ester standards were purchased from Sigma-Aldrich Chemical (Sigma, St Louis, MO, United States), and methanol was purchased from Merck (Merck, Darmstadt, Germany). The *N*-acetyl-6-methoxytryptamine standard was purchased from FUJIFILK Wako Pure Chemical Corporation (FUJIFILK Wako, Tokyo, Japan).

### Detection, Identification and Quantitative Analyses of Melatonin and Its Isomers

Melatonin was extracted according to the modified method of Vitalini et al. and others (Chen et al., 2003; Vitalini et al., 2013; Wang et al., 2016). Briefly, 5 g of oven-dried or freeze-dried sample was accurately weighed and transferred to a 50-mL centrifuge tube. A 10-mL aliquot of methanol was added to each sample tube and vortexed for 2 min. Ultrasonication in an ultrasonic water bath (200 W, 20°C) followed by 30 min on ice was used to assist and accelerate the extraction of melatonin. After centrifugation at 12,000 × *g* for 5 min at 4°C, the supernatants were collected and filtered through a 0.22-μm syringe filter and stored in amber vials suitable for subsequent UPLC-MS/MS analyses (Pothinuch and Tongchitpakdee, 2011; Wang et al., 2016). All the samples were analyzed using an Agilent 1290-6495 UPLC-MS/MS (Agilent, Waldbronn, Germany). Each sample was tested on a C18 column (2.1 cm × 5.0 cm, 1.8 μm) using the following parameters: ion source, AJS-ESI +; acquisition mode, MRM mode; dry gas temperature, 250°C; dry gas flow rate, 14 L/min; nitrogen pressure, 30 psi; sheath gas temperature, 375°C; sheath gas flow rate, 12 L/min; capillary voltage, 4,000 V; and nozzle voltage, 500 V. The mobile phases were 0.1% formic-acid in water (A) and MeOH (B). The gradient elution program was set as follows: 0–2 min, 10% (B); 2–4 min, 10–90% (B); 4–6 min, 90–98% (B); 6–8 min, 98–10% (B). The flow rate was set at 0.15 mL/min and the injection volume was set at 3.0 μL. The melatonin and isomers contents of each sample were determined in triplicate.

An UHPLC-Q-TOF-MS system, performed on a Waters Xevo G2-XS QTOF system equipped with a heated ESI mode and coupled to a Waters I-Class UHPLC system (Waters, Milford, CT, United States) was used for the analysis of melatonin isomers, melatonin, tryptophan-ethyl ester and samples. Each sample was tested on a BEH C18 column (2.1 cm × 5.0 cm, 1.7 μm) (Waters). The mobile phases were 0.1% formic-acid in water (A) and MeOH (B). The gradient elution program was set as follows: 0–2 min, 10–15% (B); 2–4 min, 15–30% (B); 4–8 min, 30–40% (B); 8–10 min, 40–60% (B); 10–12 min, 60–85% (B). The flow rate was set at 0.4 mL/min and the injection volume was set at 1.0 μL. ESI was operated in the positive electrospray ionization modes. The electrospray capillary voltage was 2.5 kV, the capillary skimmer was set to 40 V, a countercurrent flow of nitrogen gas (120°C) was employed for the desolvation, and argon was used to improve the fragmentation. The scan range was from *m/z* 80–400 at a resolution of 60.000.

### Quantitative Real-Time PCR Analysis

The total RNAs isolated from four mulberry leaves and fruit (“Dashi,” “Baiyuhuang,” “Jialing NO. 30,” and “Zhongsang 5801”) were extracted, independently, using RNAiso Plus (TaKaRa, Dalian, China) according to the manufacturer’s instructions. The RNA quality and concentration were measured using a ND-1000 UV spectrophotometer (Thermo, Madison, WI, United States). First-strand cDNA was synthesized using 3 μg of total RNA with M-MLV reverse transcriptase (Promega, Madison, WI, United States) in a 25 μL reaction system. The amino acid sequences encoded by 37 potential genes involved in the biosynthesis of melatonin and its isomers were downloaded from the *M. notabilis* database^1^. The primers (Supplementary Table 2) used for qRT-PCR were designed based on the gene sequences obtained from *Morus* genome. Each qRT-PCR was performed using SYBR Premix EXTaq II (TaKaRa) and StepOnePlus Real-Time PCR System (Applied Biosystems, Foster City, CA, United States) according to the manufacturer’s instructions. The diluted cDNA (2 μL) was used as a template. The mulberry *Actin3* gene (GenBank accession NO. HQ163775.1) was used as a control to normalize target gene expression data. Supplementary Table 2 contains a list of gene-specific primers for qRT-PCR. The process of detecting the expression levels of mulberry genes by qRT-PCR was as follows: 10 μL SYBR Premix ExTaq II, 0.4 μL primer-F (10 μM), 0.4 μL primer-R (10 μM), 0.4 μL ROX reference dye, 2 μL template, 6.8 μL ddH_2_O, for a 20-μL total. The reaction was as follows: 40 cycles of pre-denaturation at 95°C for 30 s, denaturation at 95°C for 5 s and extension at 60°C for 30 s.

### Cloning and Recombinant Overexpression of Key Genes Involved in Melatonin Isomer Biosynthesis

Based on the *M. notabilis* database (see text footnote 1), the sequences of the *ASMT* genes in mulberry were established. All the primer pairs (Supplementary Table 3) were designed using Premier 5.0 (Biosoft International Palo Alto, United States) to clone *MaASMT4*, *MaASMT9*, *MaASMT19* and *MaASMT20* from “DaShi” using PCR. Total RNA extraction and cDNA synthesis were performed as previously described. The PCR products were cloned into the pMD19-T cloning vector, and the inserts were verified and sequenced.

### Functional and *in vitro* Activity Analyses of the Key Enzymes Involved in Melatonin Isomer Biosynthesis

The ASMT cDNA from pMD19-*MaASMT4*, pMD19-*MaASMT9*, pMD19-*MaASMT19* and pMD19-*MaASMT20* were first subcloned into the pCold TF vector (TaKaRa, Beijing, China;^2^, Code No. 3365) by LR recombination to obtain the respective expression vectors pCold TF-*MaASMT4*, pCold TF-*MaASMT9*, pCold TF-*MaASMT19* and pCold TF-*MaASMT20* according to the instruction of *pEASY*^®^-Basic Seamless Cloning and Assembly Kit (TransGen Biotech, Beijing, China;^3^, Order NO. CU201). Next, these expression vectors were independently transformed into *Escherichia coli* BL21 (DE3). Each of the obtained positive *E. coli* strains was inoculated into a sterilized test tube, and positive *E. coli* strains and fresh medium containing 100 mg/L ampicillin were mixed at a1:100 ratio and then incubated at 37°C until an OD_600_ of 0.6. After the addition of 1 mM isopropyl-b-D-thiogalactopyranoside (+ IPTG), the culture was grown at 28°C and shaken at 250 rpm for 8 h. Cell culture and purification steps were performed using Ni-NTA chromatography according to the manufacturer’s instructions (Sangon Biotech, Shanghai, China;^4^, Order NO. C600033). The protein concentration was determined by the Bradford method using a protein assay dye (Beyotime, Shanghai, China). Purified recombinant MaASMT4, MaASMT9, MaASMT19 and MaASMT20 were dissolved in 30% glycerol and stored at −80°C or analyzed further (Byeon et al., 2014).

Samples of the purified recombinant proteins (2.0 μg) were incubated in a total volume of 100 μL of 100 mM potassium phosphate buffer (pH 7.8) containing 1 mM *N*-acetylserotonin and 0.5 mM *S*-adenosyl-L-methionine at 28°C for 30 min, and reactions were terminated by the addition of 300 μL of MeOH. Then, 10 μL aliquots were analyzed using UPLC-MS/MS as described above (Kang et al., 2011; Sangkyu et al., 2013). Non-enzymatic reaction products, which were generated in the absence of any enzyme, were subtracted. The analyses were performed in triplicate.

### Statistical Analyses

All the data were expressed as the means ± standard deviations analyzed with SPSS statistical software (SPSS Inc., Chicago, IL, United States).

## Results

### Detection and Identification of Melatonin and Its Isomers in Mulberry Leaves

**FIGURE 1:**
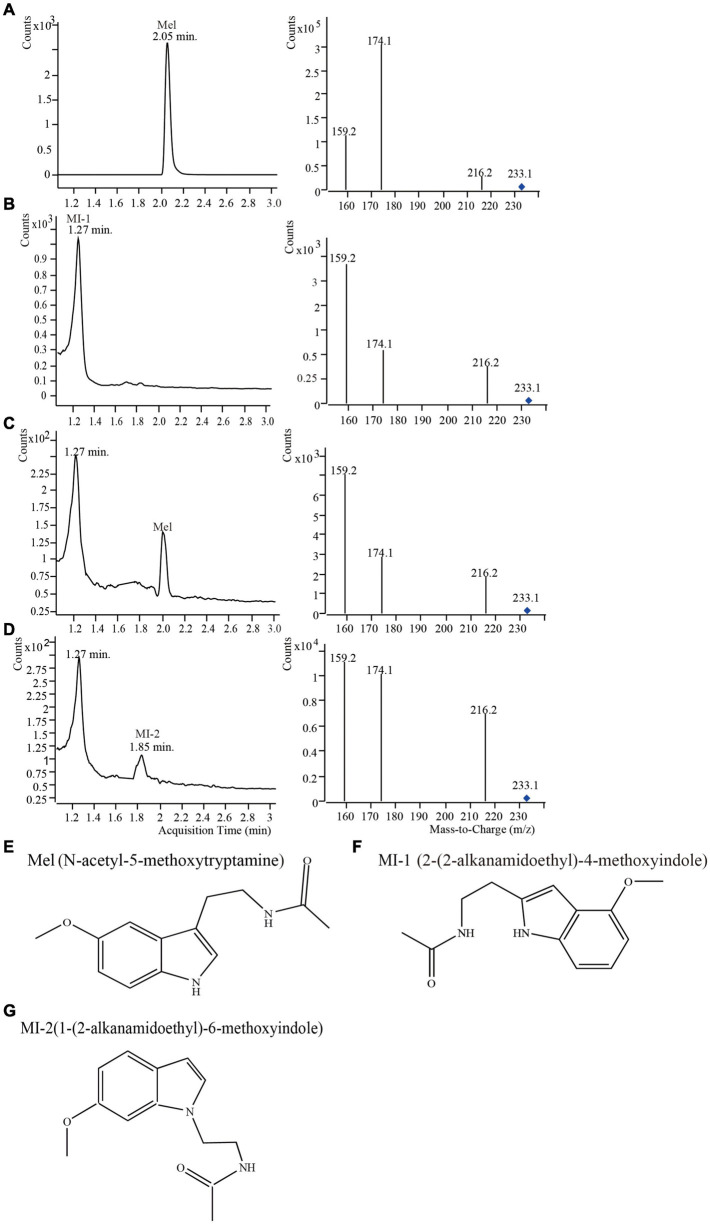
Mass spectra of mulberry leaf samples from different varieties harvested in May, 2015 detected using UPLC-MS/MS, and the structural formulae of melatonin and its isomers. **(A)** Melatonin standard; **(B)** Sample S-36-L; **(C)** Sample S-4-L; **(D)** sample S-12-L; **(E)** melatonin; **(F)** 2-(2-alkanamidoethyl)-4-methoxyindole; **(G)** 1-(2-alkanamidoethyl)-6-methoxyindole.

UPLC-MS/MS was used to determine whether the retention time and the collision-induced ion fragments of mulberry samples in positive mode were consistent with those of the melatonin standard (Figure 1). The retention time of the melatonin standard was 2.05 min (Figure 1A). The main collision-induced ion fragments derived from the melatonin standard were 233.0, 216.2, 174.1, and 159.2. In melatonin collision-induced ion fragments, the relative abundance of 174.1 was greatest, which indicated that the most stable abundant fragment of melatonin was 174.1 (Figure 1A). Therefore, the collision-induced ion fragment of 174.1 was used for the quantification of melatonin in accordance with a previous report (Rodrigueznaranjo et al., 2011). We detected three compounds with the same *m/z* as melatonin using UPLC-MS/MS (Figures 1B–D). The three compounds were named as Mel, MI-1 and MI-2, respectively. The Mel were detected at 2.05 min and the same *m/z* value (233.0) as that of the melatonin standard. These data indicated that Mel was melatonin (Figure 1C). In addition, Compared to the melatonin standard, the two natural compounds (MI-1 and MI-2) have the same *m/z* value (233.0) and different retention times (1.27 and 1.85 min), and the most abundant fragment of the MI-1 in mulberry leaves occurred at 159.2 (Figures 1B–D). Diamantini et al. (1998) previously reported the characterization of metastable collision-induced ion fragments of melatonin isomers. The data showed that the different positions of the substituents on the indole ring influence the fragmentation patterns and the relative abundances of the generated ions (Tan et al., 2012). Therefore, MI-1 and MI-2 were thought to be melatonin isomers, and the collision-induced ion fragment of 159.2 was used for the quantification of MI-1 (Gomez et al., 2012). MI-2 was only present in four mulberry varieties, and the low content made it impossible to quantify. The structure of Mel and the predicted structures of MI-1 and MI-2 are shown in Figures 1E–G and based on the different collision-induced ion fragment patterns (Diamantini et al., 1998; Gomez et al., 2012).

### Analysis of Melatonin and Its Isomers in Different Mulberry Varieties

The contents of melatonin and its isomers from the 50 mulberry varieties are shown in Table 1, and 42 varieties only contained the novel natural melatonin isomer in their leaves. One mulberry variety only contained melatonin and the remaining seven mulberry varieties contained both melatonin and its isomers, with their total contents ranging from 0.23 to 20.58 ng/g (DW). The highest melatonin content was detected in the sample from S2-L (0.83 ng/g DW) and the highest melatonin isomer content was detected in the sample from S50-L (20.58 ng/g DW) (Table 1). The content of the melatonin isomers ranged from 0.23 to 20.58 ng/g (DW). The average content of melatonin isomers in mulberry leaves was 5.49 ng/g (DW) (Table 1).

### Spatio-Temporal Distributions of Melatonin and Its Isomers in Mulberry

To uncover the effects of daily environmental conditions (season) on the production of melatonin and its isomers in mulberry leaves, leaves were collected every 2 months at harvest day. The changes in the content of melatonin and its isomer in three mulberry varieties were different in different months (Supplementary Figure 1). The melatonin isomer contents in mulberry leaves at the same developmental stage from different varieties increased from April 28th to June 28th, but showed the opposite trend from June 28th to August 28th. The average melatonin isomer content in S44-L was higher than that in S4-L and S26-L at different harvest time points.

**FIGURE 2:**
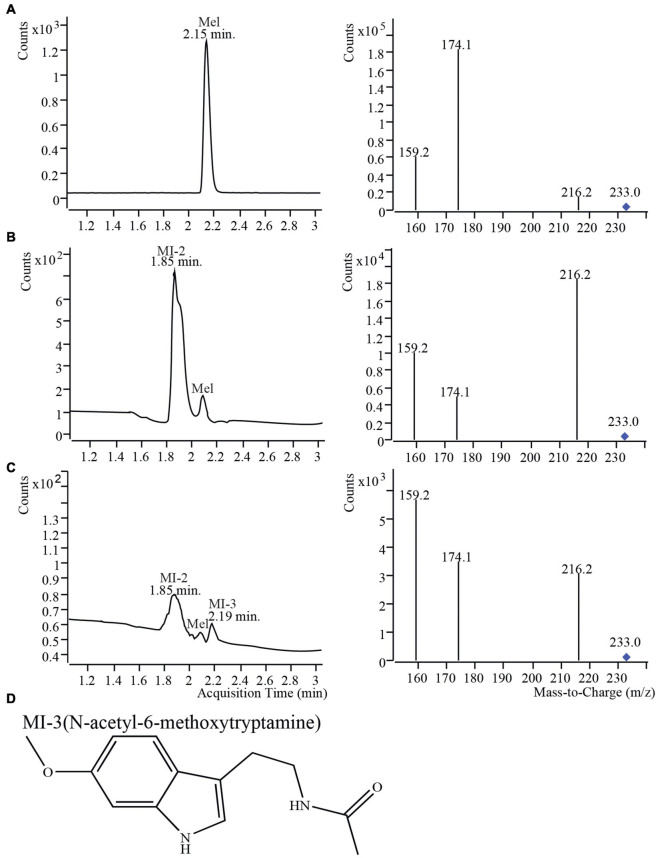
Mass spectra of mulberry leaf and fruit samples from “Dashi” harvested in 2017 detected using UPLC-MS/MS. **(A)** Melatonin standard; **(B)** Mulberry fruit; **(C)** Mulberry leaves; **(D)** The structure of a melatonin isomer. MI-3, *N*-acetyl-6-methoxytryptamine.

To measure melatonin and its isomer in the leaves of S4-L, S26-L and S44-L at different maturation stages, leaves at different positions (1st, 5th, 10th, 15th, and 20th) were collected. In the three mulberry varieties, the MI-1 content was higher at the 20th leaf position than at the other leaf positions (Supplementary Figure 2). The melatonin isomer contents in leaves at different maturation stages were significantly different in S4-L, S26-L and S44-L (*P* < 0.05). Thus, the melatonin isomer contents in the leaves of three different mulberry varieties increased with leaf position. However, the levels of melatonin and its isomer were below the detection limits in the 1st leaves of the three mulberry varieties. In general, the melatonin isomer contents increased with leaf position. To our knowledge, this is the first report of melatonin isomers being naturally present in the tissues of a woody plant. In addition, although melatonin isomers do exist in fermentation products and herbaceous plants, the biosynthesis of melatonin isomer was still completely unclear (Gomez et al., 2013). Therefore, we conducted a series of experiments using the same materials to clarify the molecular mechanism underlying the biosynthesis of melatonin and its isomers in mulberry. Melatonin and isomers from different tissues (leaves, fruit) of four different mulberry varieties were detected using UPLC-MS/MS (Figure 2 and Supplementary Table 1). As shown in Figure 2, the retention time of the melatonin standard was 2.15 min and contained mainly three ion fragments, 216.2, 174.1, and 159.2. The relative abundance of the main ion fragments of melatonin standard was highest at 174.1 (Figure 2A). Three compounds having *m/z* 233.0, the same *m/z* as the melatonin standard, were detected at retention times of 1.85, 2.15 and 2.19 min (Figures 2B,C). The three compounds were named as Mel, MI-2 and MI-3 in accordance with a previous method. Mel showed the same *m/z* value (233.0) and retention time as the melatonin standard (Figure 2B). The relative abundances of the main fragment ions of the samples (216.2 or 159.2) were inconsistent with those of the melatonin standard (174.1). Thus, MI-2 and MI-3 were identified as melatonin isomers (Figures 2B,C). As an authentic marker is not available to identify the current isomer detected by UPLC-MS/MS, a tentative identification was performed by comparing relative abundances of minority fragment ions. Previous studies have demonstrated that 216.2 is important as the primary ion, and the relative abundances of secondary ions can indicate the positions of the methoxy and amide groups (Gomez et al., 2012). The predicted structure of MI-2 is 1-(2-alkanamidoethyl)-6-methoxyindole. In accordance with previous reports, MI-3 is presumed to be *N*-acetyl-6-methoxytryptamine (Figure 2D; Diamantini et al., 1998; Gomez et al., 2013). Four different mulberry varieties contained melatonin and two melatonin isomers (MI-2 and MI-3) in their leaves. However, four different mulberry varieties fruits only contained melatonin and one melatonin isomer (MI-2). We found that MI-3 was only present in the leaves and not in the fruit of the four different mulberry varieties (Supplementary Table 1). Furthermore, the total contents of melatonin and melatonin isomers in leaves and fruits of “Dashi” were the highest among the four mulberry cultivars (Supplementary Table 1).

### Tissue-Specific Expression of Genes Involved in the Biosynthesis of Melatonin and Its Isomers

**FIGURE 3:**
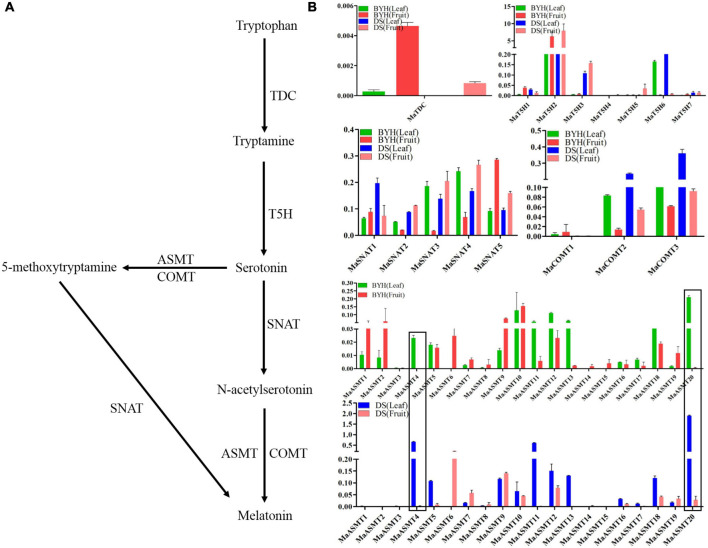
Expressional analysis of genes involved in the biosynthesis of melatonin and its isomers in two tissues (leaf, fruit) of two mulberry varieties sampled in 2017. **(A)** Biosynthetic pathway of melatonin. **(B)** The expression profiles of genes involved in the biosynthesis of melatonin and its isomers. BYH represents the “Baiyuhuang” variety; DS represents the “Dashi” variety. Data are means ± SDs (*n* = 3).

The complete sequence of the mulberry genome is now available (He et al., 2013; Jiao et al., 2020). It provides the opportunity to elucidate the molecular mechanisms underlying the biosynthesis of melatonin and its isomers. Based on the analysis of the mulberry genome database, 37 potential genes, one *TDC*, seven *T5Hs*, six *SNAT*s, three *COMT*s, and 20 *ASMT*s, were identified as being associated with the biosynthesis of melatonin (Zheng et al., 2021). The qRT-PCR was used to detect the expression levels of genes involved in the biosynthesis of melatonin in leaves and fruit of mulberry varieties “Dashi” and “Baiyuhuang” (Figures 3A,B). All the genes involved in the biosynthesis of melatonin, except *MaASMT4* and *MaASMT20*, had different expression patterns in both the leaves and fruit of the two varieties (Figure 3B). *MaTDC* was expressed in various tissues, and the gene expression in fruit was significantly higher than that in leaves (Figure 3B). *MaT5H2* was expressed highest in these two tissues compared with the expression levels of six other genes (Figure 3B). The expression levels of the six *MaSNAT* genes differed in two tissues of two varieties, but their expression patterns were consistent (Figure 3B). Among the three *MaCOMT* genes, the expression level of *MaCOMT1* was the highest (Figure 3B). The expression levels of *ASMT* gene family members were different in the two varieties (Figure 3B). The *MaASMT4* gene was abundantly expressed in the leaves of “Dashi” and “Baiyuhuang,” but was almost not expressed in the fruit of the two mulberry varieties. The expression level of the *MaASMT20* gene in the leaves of the two mulberry varieties was over 25-fold greater than in their fruit (Figure 3B). A similar result was confirmed in the other two mulberry varieties (“Jialing NO. 30” and “Zhongsang 5801”) (Supplementary Figure 3). These results suggested that *MaASMT4* and *MaASMT20* may be involved in the synthesis of melatonin isomer (MI-3), which was only detected in mulberry leaves.

### Gene Cloning and Analysis of the *MaASMT4* and *MaASMT20*

**FIGURE 4:**
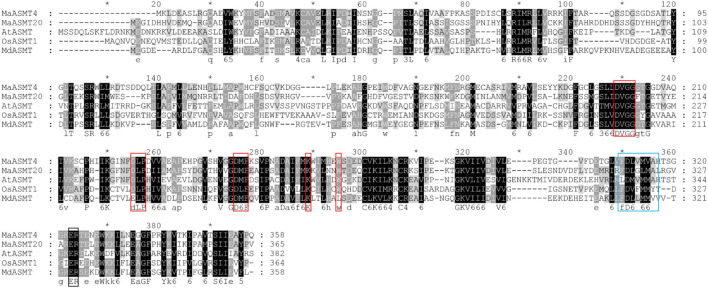
Multiple sequences alignment of MaASMTs with the ASMTs from other plant species. The positions of the different conserved domains are represented by different colored boxes. The conserved motif for the *S*-adenosine-L-methionine binding is boxed in red, putative substrate-binding residues are boxed in black, and catalytic residues are boxed in blue.

We successfully cloned *MaASMT4* and *MaASMT20* from “Dashi.” The cloned *MaASMT4* was 1,077 bp in length, encoding 358 amino acids, while the cloned *MaASMT20* was 1,098 bp in length, encoding 365 amino acids (GenBank accession NOs. MN937267 and MN937270). The amino acid sequences of MaASMT4 and MaASMT20 were aligned with AtASMT, MdASMT and OsASMT1 (Figure 4). The conserved motif for *S*-adenosyl-L-methionine binding sites in the MaASMT4 and MaASMT20 sequences were identical to those of the AtASMT, MdASMT and OsASMT sequences. The conserved motif for the putative substrate binding sites of MaASMT4 and MaASMT20 were also identical to those of AtASMT, MdASMT and OsASMT, but the catalytic domains were not conserved.

### Function and Activity Analyses of *MaASMT4* and *MaASMT20*

**FIGURE 5:**
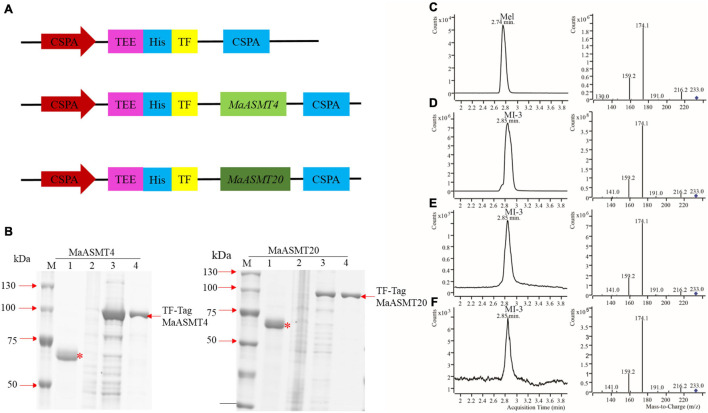
Expression and activity analyses of MaASMT4 and MaASMT20 proteins. **(A)** Schematic diagram of the *E. coli* expression vector in pCold TF harboring *MaASMT4* and *MaASMT20*. **(B)** Purification of N-terminal His × 6-tagged MaASMT4 and MaASMT20 proteins. *E. coli* BL21 (DE3) cells harboring either pCold TF-*MaASMT4* or pCold TF-*MaASMT20* were incubated with IPTG for 8 h at 28°C. Lane 1: protein marker; lane 2: pCold TF (+ IPTG); lane 3: recombinant (–IPTG) lane 4: recombinant (+ IPTG); lane 5: purified protein. “TF” is Trigger Factor, a prokaryotic ribosome-related molecular chaperone that facilitates the translation and folding of peptides. “^∗^” indicates the Trigger factor protein. “–IPTG” indicates no addition of 1 mM IPTG to the *E. coli* BL21 (DE3) culture, “+IPTG” indicates addition of 1 mM IPTG to the *E. coli* BL21 (DE3) culture. Products of *in vitro* enzymatic reactions detected by UPLC-MS/MS. **(C)** Melatonin standard; **(D)** *N*-acetyl-6-methoxytryptamine standard; **(E)** Chromatogram of *N*-acetylserotonin catalyzed by MaASMT4; **(F)** Chromatogram of *N*-acetylserotonin catalyzed by MaASMT20.

While the animal ASMT enzyme has been characterized in detail at the biochemical level, there have been limited studies of plant ASMT enzymes. *MaASMT4* and *MaASMT20* full-length cDNAs were independently cloned into pCold TF vectors for the expression of recombinant proteins in *E. coli* (Figure 5A). We purified recombinant proteins as described in the Materials and Methods. The obtained MaASMT4 and MaASMT20 proteins showed the predicted molecular weights of 39 and 41 kDa, respectively, in the SDS-PAGE analysis (Figure 5B). Then, we co-incubated *S*-adenosyl-L-methionine and *N*-acetylserotonin separately with MaASMT4 and MaASMT20 in the reaction buffer and analyzed the products by UPLC-MS/MS (Figures 5C–F). The retention time for the melatonin standard was 2.81 min and that of the MI-3 standard was 2.91 min (Figures 5C,D). The target compound in both the MaASMT4 and MaASMT20 reaction samples showed the same *m/z* value (233.0) and the same retention time (2.91 min) as that of MI-3 standards standard (Figures 5C–F). These data indicated that MaASMT4 and MaASMT20 were able to convert *N*-acetylserotonin to melatonin and MI-3.

## Discussion

In this study, the contents of melatonin and its isomers were analyzed among 50 mulberry varieties and they differed significantly by up to 90-fold. The measured melatonin content is affected by genetic and non-genetic factors (Wang et al., 2016). The non-genetic factors include growth conditions and analytical methods, such as LC-ECD-UV, LC-MS, and LC-MS/MS (Zhao et al., 2013; Mukherjee et al., 2014; Wang et al., 2016). The variation in the contents of melatonin and its isomers in mulberry leaves of different varieties may mainly depend on genetic traits, because samples at the same maturity stage were collected from mulberry trees that were grown under the same environmental conditions and melatonin was measured using the same analytical methods. The melatonin contents of mulberry leaves from the three other reports differed greatly, which may be attributed to the selectivity and sensitivity of different analytical methods for the identification and quantification of melatonin (Chen et al., 2003; Pothinuch and Tongchitpakdee, 2011; Wang et al., 2016). The total content of melatonin and melatonin isomers in this study was similar to the results reported by Wang et al. (2016). Our measurement of melatonin in mulberry may be more reliable because a more precise analytical method, UPLC-MS/MS, was used.

Although some studies have used MS to detect melatonin in plants, little attention has been paid to the detection and characterization of melatonin isomers (Iriti et al., 2006; Feng et al., 2014; Vitalini et al., 2020). Here, natural compounds (MI-1, MI-2, and MI-3) having the same *m/z* as melatonin, were found in mulberry using UPLC-MS/MS. MI-1, MI-2 and MI-3 were thought to be melatonin isomers. Melatonin isomers have been detected in wine, and one of these isomers has been identified as tryptophan-ethyl ester, a compound with the same *m/z* as melatonin standard (Gardana et al., 2014; Iriti and Vigentini, 2015). However, UHPLC-Q-TOF-MS was carried out to confirm that in mulberry none of these compounds is tryptophan-ethyl ester but were other melatonin isomers. Tryptophan-ethyl ester has the same *m/z* as melatonin standard but its retention time was inconsistent with those of the two natural compounds (Supplementary Table 4). Therefore, the novel natural compounds in mulberry leaves were not tryptophan-ethyl ester. To our knowledge, this is the first identification of novel melatonin isomers in a woody plant, and this lays the foundation for the further clarification of the biosynthetic pathway of melatonin and its isomers in plants.

To our knowledge, the physiological functions of melatonin isomers in plants are completely unknown. However, Tarzia et al. (1997) tested the biological activity and the ability of a synthetic melatonin isomer (A1/M6) to bind to melatonin membrane receptors. Spadoni et al. (2001) reported that changing the positions of either the methoxy or *N*-acetylaminoethyl side chains in the indole ring results in marked alterations in their antioxidant and cytoprotective capacities (Tarzia et al., 1999). For example, if the methoxy side chain is located in position 4 of the indole ring, then this melatonin isomer possesses the strongest antioxidant capacity, but if the *N*-acetylaminoethyl side chain is in position 3, then the isomer is most effective as a cytoprotective agent (Spadoni et al., 2001). Therefore, we initially speculated that melatonin and its isomers in mulberry play similar functions during plant growth and development.

**FIGURE 6:**
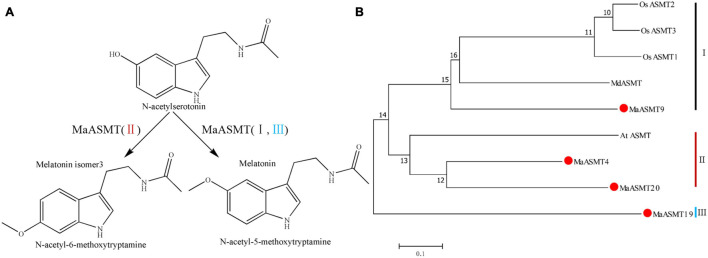
The final step of the biosynthetic pathways of melatonin and its isomer and a phylogenetic tree of the ASMT family. **(A)** The final step of the biosynthetic pathways of melatonin and its isomer in “Dashi.” ASMT: *N*-acetylserotonin methyltransferase. **(B)** Phylogenetic tree of amino acid sequences of ASMT in “Dashi” and other plants. The phylogenetic tree was generated with the neighbor-joining method using MEGA5 software. ASMTs were separated into three groups: class I (black), II (red) and III (blue). The function of ASMT genes has been characterized. Information of ASMT genes was listed in Supplementary Table 5.

In plants, several isoforms of SNAT and ASMT have been identified in apple and rice (Kang et al., 2011; Bixiao et al., 2015; Tan and Reiter, 2020). The activity levels of some of these isoforms do not always correlate with the production of melatonin. Whether some of the SNAT and ASMT isoforms are actually enzymes for the synthesis of melatonin isoforms is unknown (Yeong et al., 2013). ASMT has three groups, classes I, II and III (Sangkyu et al., 2013; Liu et al., 2017; Pan et al., 2019). Our result suggested that *MaASMT4* and *MaASMT20*, belonging to the class II *ASMT* gene family, were the key genes for the biosynthesis of melatonin isomer, MI-3, which only occurs in mulberry leaves (Figures 5, 6A). *ASMT* genes of rice and apple belong to class I, and *ASMT* gene of *Arabidopsis* belong to class II (Figure 6B). The proteins encoded by these genes only catalyze the conversion of *N*-acetylserotonin to melatonin (Sangkyu et al., 2013; Byeon et al., 2016). Therefore, this indicated that the functions of the *MaASMT4* and *MaASMT20* genes were different from those of other *ASMT* genes. In addition, the other two *MaASMTs* could be used in *in vitro* enzyme activity experiments. They were class I member MaASMT9 and class III member MaASMT19 (Figure 6B). Thus, MaASMT9 (GenBank accession NO. MN937268) and MaASMT19 (GenBank accession NO. MN937269) were also cloned from “Dashi.” MaASMT9 and MaASMT19 full-length cDNAs were independently cloned into pCold TF vectors for the expression of recombinant proteins in *E. coli* BL21 (DE3) (Supplementary Figure 4A). The MaASMT9 and MaASMT19 proteins were purified according to description in the Materials and Methods, and the obtained proteins were with the predicted molecular weights of 41 kDa and 40 kDa, respectively (Supplementary Figure 4B). We co-incubated *S*-adenosyl-L-methionine and *N*-acetylserotonin separately with MaASMT9 and MaASMT19 in the reaction buffer and analyzed the products by UPLC-MS/MS. The recombinant proteins of the two MaASMTs (classes I and III) were only able to convert *N*-acetylserotonin to melatonin (Supplementary Figures 4C–F).

Why the ASMT proteins perform distinct functions? We analyzed sequences of ASMTs that contained conserved OMT domains in plants, including *S*-adenosyl-L-methionine binding sites, substrate binding sites and catalytic sites (Supplementary Figure 5). The conserved motif for the *S*-adenosyl-L-methionine binding sites and substrate binding sites were well conserved among MaASMTs, AtASMT, MdASMT, and OsASMT1. However, the conserved motif in the catalytic domains of the seven ASMT proteins was not conserved. The variation among the catalytic domains may cause changes in the functions of the MaASMT enzymes belonging to class II of the ASMT family. The molecular mechanisms involved in their functional changes need to be further studied.

In conclusion, we were the first to discover three naturally occurring melatonin isomers in mulberry. In addition, MI-3, one of the three melatonin isomers was only found in mulberry leaves, was synthesized by two enzymes, MaASMT4 and MaASMT20, belonging to the class II ASMT protein family. The present study provides some new insights into the functions of MaASMT4 and MaASMT20 from the class II subgroup of the ASMT family in mulberry plants and lays a foundation for the further understanding of the biosynthesis of melatonin and its isomers. Unfortunately, the biosynthetic mechanism responsible for the higher levels of MI-1 and MI-2 in mulberry have not yet been clarified. Therefore, the analysis of the biosynthesis mechanism of MI-1 and MI-2 needs be conducted in further research.

## Data Availability Statement

The datasets presented in this study can be found in online repositories. The names of the repository/repositories and accession number(s) can be found in the article/Supplementary Material.

## Author Contributions

ShaZ and AZ designed the research and wrote the manuscript. CL, MY, SW, and WF helped in the preparation for experiments materials. YZ and ShuZ conducted experiments and analyzed the data. AZ and ZX provided the financial aid for the research. All authors contributed to the article and approved the submitted version.

## Conflict of Interest

The authors declare that the research was conducted in the absence of any commercial or financial relationships that could be construed as a potential conflict of interest.

## Publisher’s Note

All claims expressed in this article are solely those of the authors and do not necessarily represent those of their affiliated organizations, or those of the publisher, the editors and the reviewers. Any product that may be evaluated in this article, or claim that may be made by its manufacturer, is not guaranteed or endorsed by the publisher.

## Footnotes

1
http://morus.swu.edu.cn/morusdb/

2
https://www.takarabiomed.com.cn/

3
https://www.transgen.com.cn/

4
https://www.sangon.com/

## Supplementary Material

Supplementary Figure 1 Contents of melatonin and its isomers in the mature mulberry leaves from three varieties harvested during different months in 2016. Melatonin and its isomers were measured using UPLC-MS/MS. 1, April 28th; 2, June 28th; 3, August 28th. Significant differences (*P* < 0.05) are marked with different letters above the bars.

Supplementary Figure 2 Contents of melatonin and its isomers in mulberry leaves at different maturity stages from three varieties harvested during July, 2016. Melatonin and its isomers were measured using UPLC-MS/MS. 1, 1st leaves; 2, 5th leaves; 3, 10th leaves; 4, 15th leaves; 5, 20th leaves. Significant differences (*P* < 0.05) are marked with different letters above the bars.

Supplementary Figure 3 Expressional analysis of *ASMT* genes in two tissues (leaf and fruit) of two mulberry varieties sampled in 2017. **(A)** The expression profiles of the *MaASMT*s in two tissues of two mulberry varieties. Sample names are shown above the heat maps. Color scale indicates the degree of expression: green, low expression; red, high expression. **(B)** Expressional analysis of *MaASMT4* and *MaASMT20*. JL-NO30 represents the “Jialing NO. 30” variety; ZS-5801 represents the “Zhongsang 5801” variety. Significant differences (*P* < 0.01) are marked with different asterisks above the bars.

Supplementary Figure 4 Expression and activity analyses of MaASMT9 and MaASMT19 proteins. **(A)** Schematic diagram of the *E. coli* expression vector in pCold TF harboring genes of interest. **(B)** Purification of N-terminal His × 6-tagged MaASMT9 and MaASMT19 proteins. *E. coli* BL21 (DE3) cells harboring either pCold TF-*MaASMT9* or pCold TF-*MaASMT19* were incubated with IPTG for 8 h at 28°C. Lane 1: protein marker; lane 2: pCold TF (+ IPTG); lane 3: recombinant (−IPTG) lane 4: recombinant (+ IPTG); lane 5: purified protein. Products of *in vitro* enzymatic reactions were detected by UPLC-MS/MS. “TF” is Trigger Factor, a prokaryotic ribosome-related molecular chaperone that facilitates the translation and folding of peptides. “^∗^” indicates the Trigger Factor. “−IPTG” indicates no addition of 1 mM IPTG to the *E. coli* BL21 (DE3) culture, “+IPTG” indicates the addition of 1 mM IPTG to the *E. coli* BL21 (DE3) culture. Sequence data have been deposited with GeneBank (Accession NOs are MN937268 for MaASMT9 and MN937269 for MaASMT19). **(C)** melatonin standard; **(D)** melatonin isomer standard (MI-3); **(E)** Chromatogram of *N*-acetylserotonin catalyzed by MaASMT9; **(F)** Chromatogram of *N*-acetylserotonin catalyzed by MaASMT19.

Supplementary Figure 5 Sequence analysis of ASMT proteins in plants. The alignment was performed using GeneDoc and the positions of the different conserved domains are represented by different colored boxes. The conserved motif for the *S*-adenosine-L-methionine binding are boxed in red, putative substrate-binding residues are boxed in black, and catalytic residues is boxed in blue.
