# Supplementary figures and images for: Molecular Mechanisms Underlying the Biosynthesis of Melatonin and Its Isomer in Mulberry
Source: Front Plant Sci. 2021 Oct 6;12:708752. doi: 10.3389/fpls.2021.708752 (PMC8526549; doi:10.3389/fpls.2021.708752)

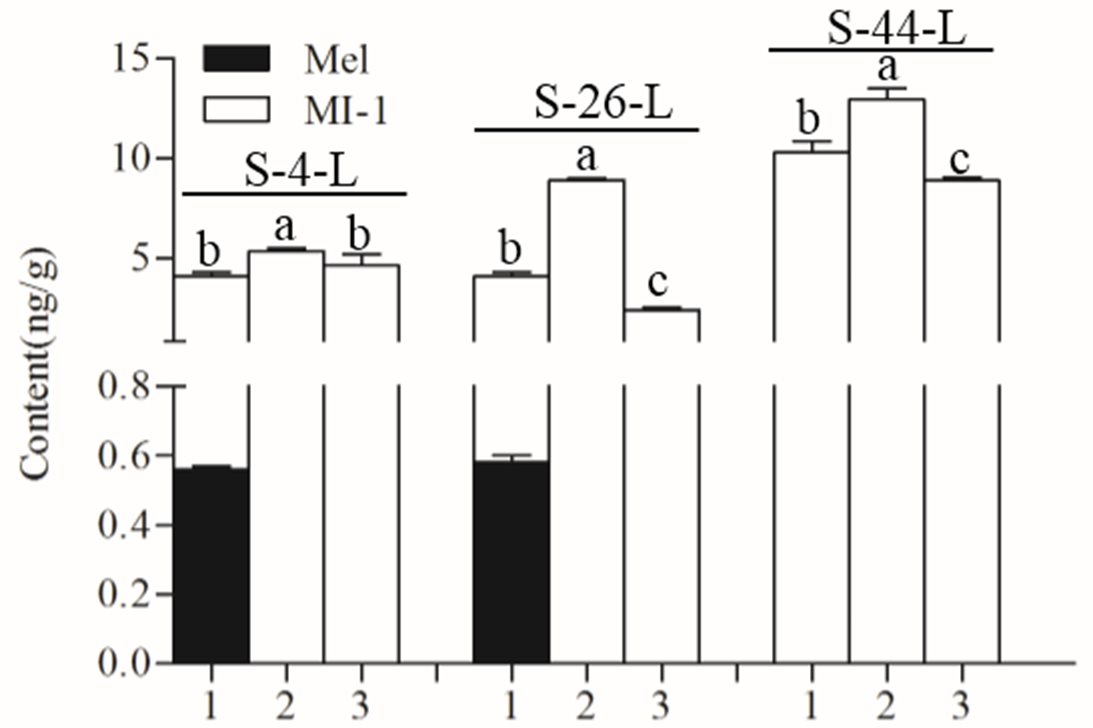

Supplement: Supplementary Figure 1 — Contents of melatonin and its isomers in the mature mulberry leaves from three varieties harvested during different months in 2016. Melatonin and its isomers were measured using UPLC-MS/MS. 1, April 28th; 2, June 28th; 3, August 28th. Significant differences (P < 0.05) are marked with different letters above the bars. [file Image_1.TIF]

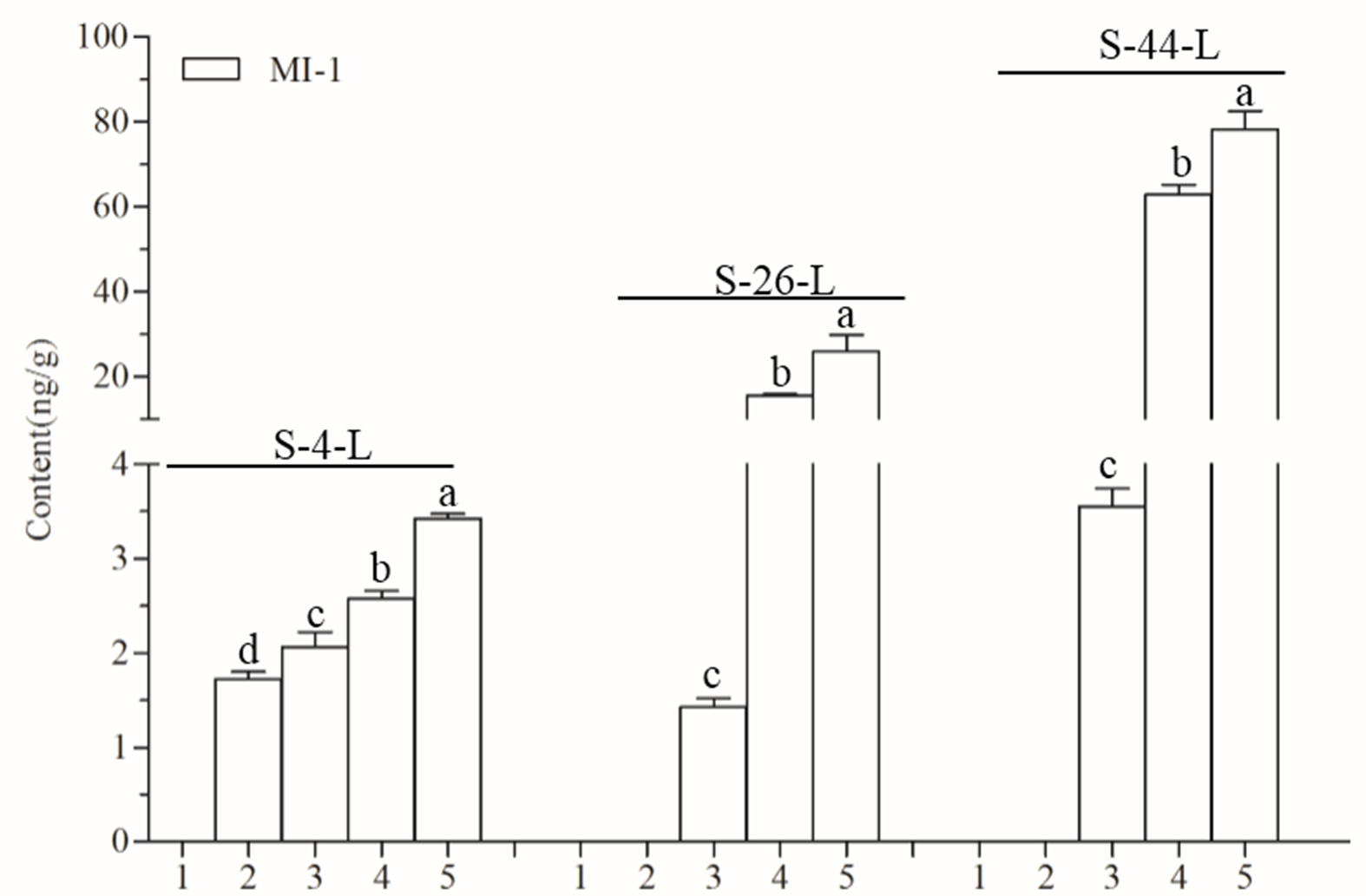

Supplement: Supplementary Figure 2 — Contents of melatonin and its isomers in mulberry leaves at different maturity stages from three varieties harvested during July, 2016. Melatonin and its isomers were measured using UPLC-MS/MS. 1, 1st leaves; 2, 5th leaves; 3, 10th leaves; 4, 15th leaves; 5, 20th leaves. Significant differences (P < 0.05) are marked with different letters above the bars. [file Image_2.TIF]

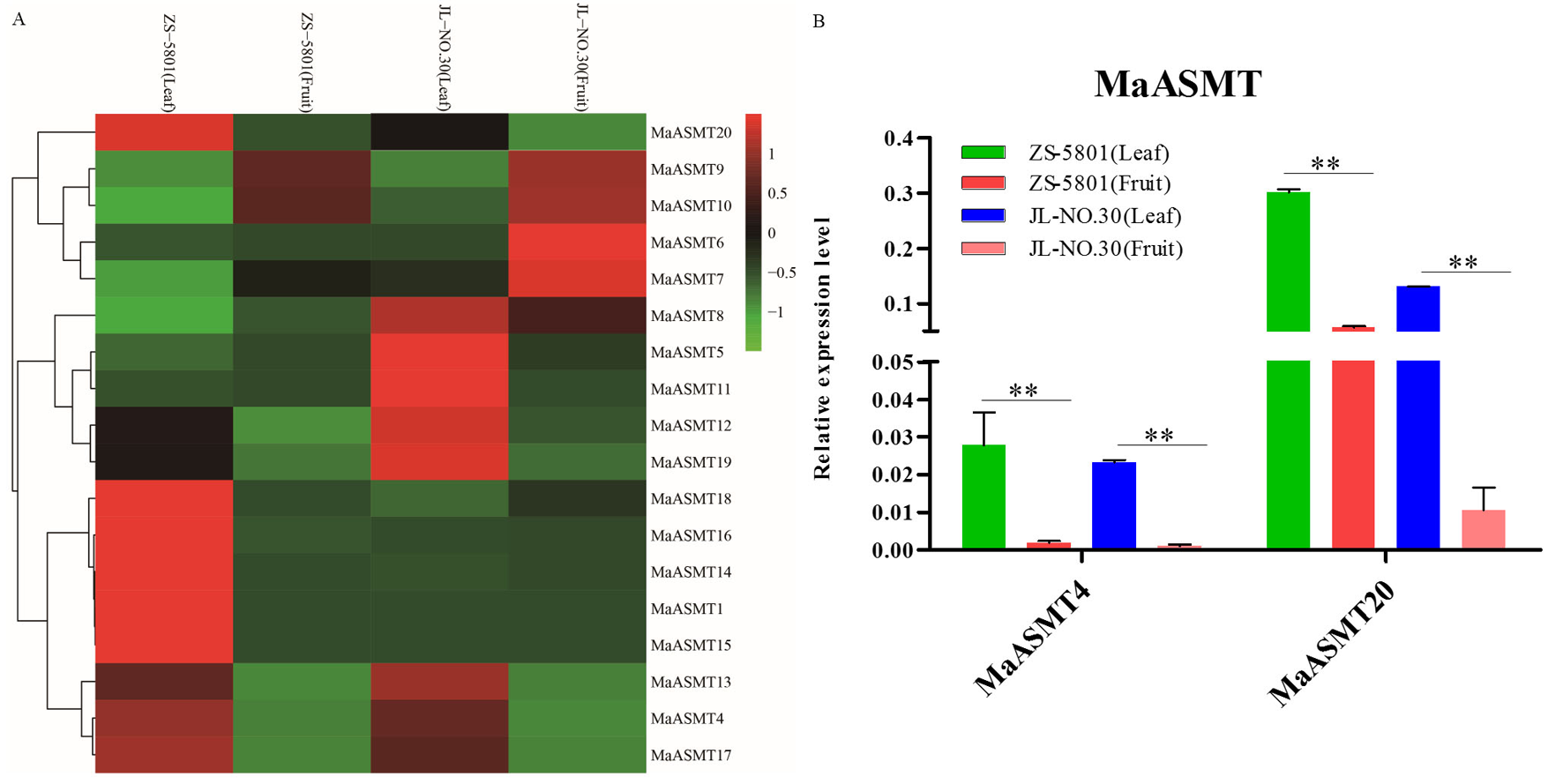

Supplement: Supplementary Figure 3 — Expressional analysis of ASMT genes in two tissues (leaf and fruit) of two mulberry varieties sampled in 2017. (A) The expression profiles of the MaASMTs in two tissues of two mulberry varieties. Sample names are shown above the heat maps. Color scale indicates the degree of expression: green, low expression; red, high expression. (B) Expressional analysis of MaASMT4 and MaASMT20. JL-NO30 represents the “Jialing NO. 30” variety; ZS-5801 represents the “Zhongsang 5801” variety. Significant differences (P < 0.01) are marked with different asterisks above the bars. [file Image_3.TIF]

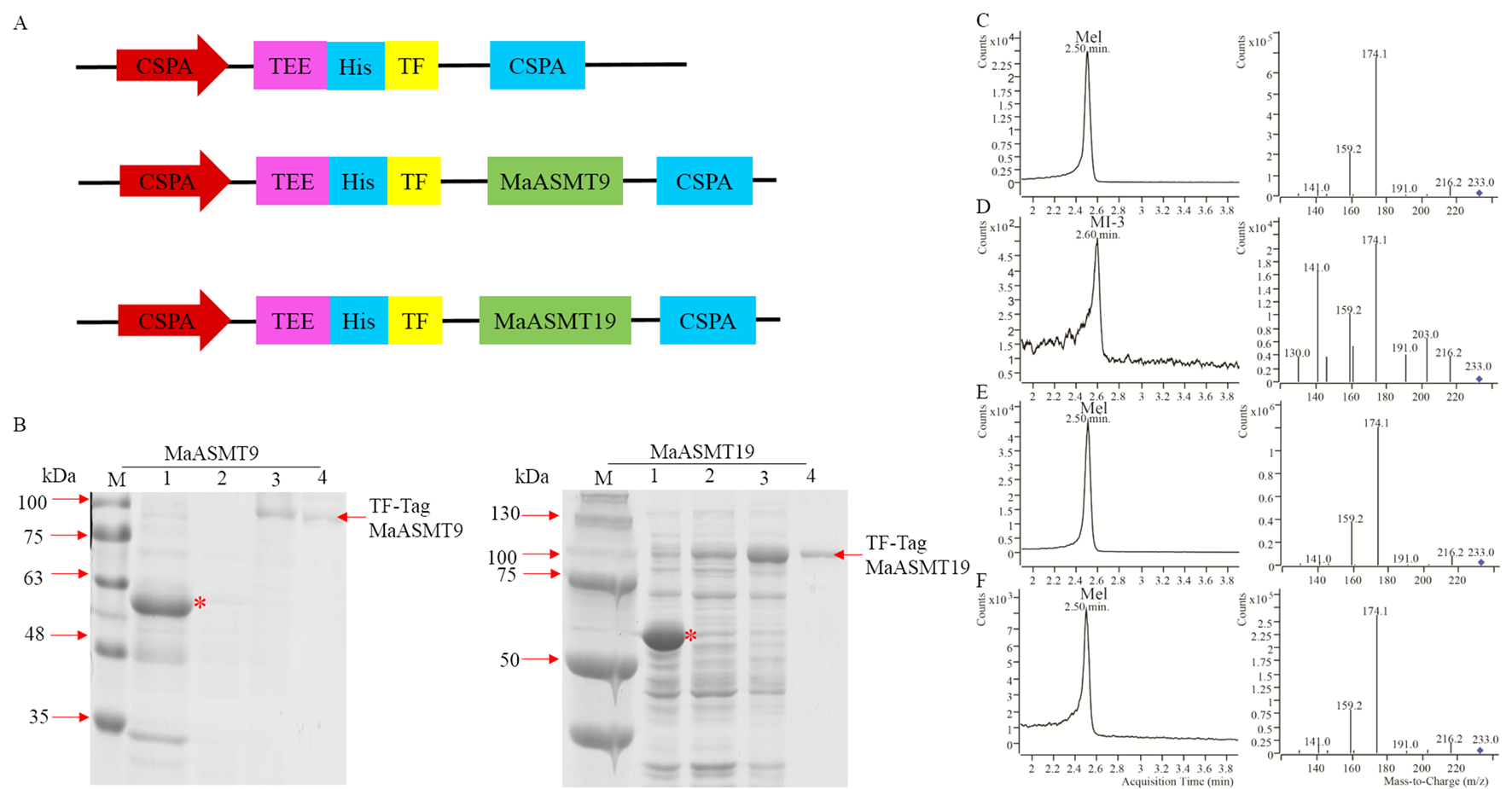

Supplement: Supplementary Figure 4 — Expression and activity analyses of MaASMT9 and MaASMT19 proteins. (A) Schematic diagram of the E. coli expression vector in pCold TF harboring genes of interest. (B) Purification of N-terminal His × 6-tagged MaASMT9 and MaASMT19 proteins. E. coli BL21 (DE3) cells harboring either pCold TF-MaASMT9 or pCold TF-MaASMT19 were incubated with IPTG for 8 h at 28°C. Lane 1: protein marker; lane 2: pCold TF (+ IPTG); lane 3: recombinant (−IPTG) lane 4: recombinant (+ IPTG); lane 5: purified protein. Products of in vitro enzymatic reactions were detected by UPLC-MS/MS. “TF” is Trigger Factor, a prokaryotic ribosome-related molecular chaperone that facilitates the translation and folding of peptides. “∗” indicates the Trigger Factor. “−IPTG” indicates no addition of 1 mM IPTG to the E. coli BL21 (DE3) culture, “+IPTG” indicates the addition of 1 mM IPTG to the E. coli BL21 (DE3) culture. Sequence data have been deposited with GeneBank (Accession NOs are MN937268 for MaASMT9 and MN937269 for MaASMT19). (C) melatonin standard; (D) melatonin isomer standard (MI-3); (E) Chromatogram of N-acetylserotonin catalyzed by MaASMT9; (F) Chromatogram of N-acetylserotonin catalyzed by MaASMT19. [file Image_4.TIF]

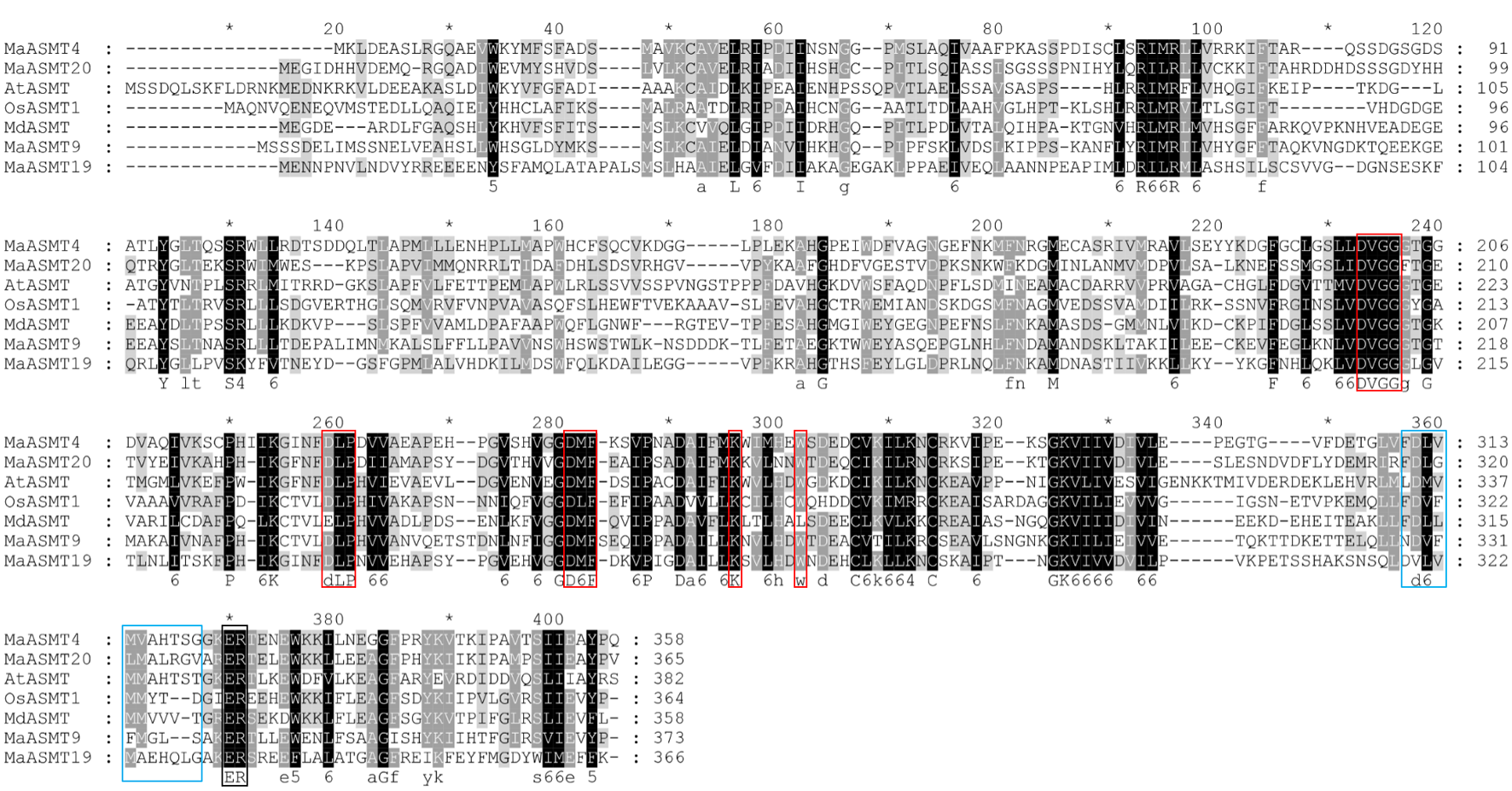

Supplement: Supplementary Figure 5 — Sequence analysis of ASMT proteins in plants. The alignment was performed using GeneDoc and the positions of the different conserved domains are represented by different colored boxes. The conserved motif for the S-adenosine-L-methionine binding are boxed in red, putative substrate-binding residues are boxed in black, and catalytic residues is boxed in blue. [file Image_5.TIF]
