# Supplementary material for: Molecular Mechanisms Underlying the Biosynthesis of Melatonin and Its Isomer in Mulberry
Source: Front Plant Sci. 2021 Oct 6;12:708752. doi: 10.3389/fpls.2021.708752 (PMC8526549; doi:10.3389/fpls.2021.708752)
Supplement: Supplementary file 6 [file Data_Sheet_1.docx]

**Supporting Table**

Supplementary Table1：Contents of melatonin and its isomers in mulberry leaves and fruit harvested in 2017.

| Name of mulberry variety | Specie name of mulberry variety | Name of sample* | Mel(ng/g)  (DW) | MI-2(ng/g)  (DW) | MI-3(ng/g)  (DW) | SUM(ng/g)  (DW) |
| --- | --- | --- | --- | --- | --- | --- |
| Jialing-NO.30 | *Morus multicaulis* Perr*.* | S4-L | d | 0.062 | d | 0.062 |
| Zhongsang5801 | *Morus multicaulis* Perr. | S10-L | d | 0.036 | d | 0.036 |
| Dashi | *Morus atropurpurea* Roxb. | S51-L | d | 0.047 | 0.021 | 0.068 |
| Baiyuhuang | *Morus alba* Linn*.* | S52-L | d | 0.057 | d | 0.057 |
| Jialing-NO.30 | *Morus multicaulis* Perr*.* | S4-F | 0.044 | 0.79 | nd | 0.834 |
| Zhongsang5801 | *Morus multicaulis* Perr. | S10-F | 0.035 | 0.95 | nd | 0.985 |
| Dashi | *Morus atropurpurea* Roxb. | S51-F | 0.061 | 0.99 | nd | 1.051 |
| Baiyuhuang | *Morus alba* Linn*.* | S52-F | 0.019 | 0.37 | nd | 0.389 |

Note： *The nomenclature of Name of sample is S for Sample, Arabic numerals for the number of mulberry variety, and the last capital letter for tissue of mulberry. For example, S1-L is S for Sample, 1 for the number of mulberry variety ‘Jialing NO. 30’, L for leaf of mulberry variety ‘Jialing NO. 30’ and F for fruit of mulberry variety ‘Jialing NO. 30’. d: detected, nd: no detected. Data are means ± SDs (*n* = 3).

Supplementary Table2. The primers used for qRT-PCR in mulberry.

| Name of gene | Forward (5' - 3') | Reverse (5' - 3') |
| --- | --- | --- |
| *MaTDC* | AACATTCGGTCGATCCAGAC | ACCGTAGCGCAGAGGTAGAG |
| *MaT5H1* | TATTCCCTTTGGTGCTGGTC | TTTGACCAGGAGGCAACTTC |
| *MaT5H2* | CAAGGGACAGGATTTTGAGC | TCCCAATCGAAGCTATGGAG |
| *MaT5H3* | GCTGCAGGAACAGACACAAC | TTCTCTCTCCCCCACAACAC |
| *MaT5H4* | GACATGGCTTTCGCCTCTTA | TGTGCAAGAGCAACTTCTGG |
| *MaT5H5* | TCCTTACTTGGGATGGCTTG | TCACCCATTCTCTGCTGATG |
| *MaT5H6* | GGTTCCTACTGGCGTGACAT | AATCGAAAGCCCAATTTCCT |
| *Ma*T5H7 | CGACCCTGATTGTTTCGTCT | AAGATATCGGTGGCTGTCGT |
| *MaSNAT1* | TGCCAAGTTTGGAATCCTTC | CTTGTGGCATTAGGCTGACA |
| *MaSNAT2* | CATGGAGCTCAAATGGATCA | AAGACGGGAAATGAGGAAGG |
| *MaSNAT3* | CATCCTCCCAACCTTCTTCC | GAGTTCAGCTGGTCGAGGTC |
| *MaSNAT4* | AGAAGAAGGAAGGCGGTTCT | AACATGGTTCAACAGCTTCG |
| *MaSNAT5* | TCAGTGGCAGCATACCAAAG | ACCAAGCTCATCTGGCAAAG |
| *MaSNAT6* | GGCACATTGAAGATGTCGTG | TTGTAACACCCGACTGAACG |
| *MaCOMT1* | CTTGCCACCAAAGGAGTTGT | GGAATCCAGAACCCTTAGCC |
| *MaCOMT2* | TTGCGTCTCTTCGCTTCATA | AACTTCGCGGTAGGTGTGAG |
| *MaCOMT3* | CTTCCAGTCGAGGCAGAAAC | AATTGTTGCGTGCTCCTTTC |
| *MaASMT1* | CATCTTGCTTACCGTGCTTG | ATGTGTTGTGGCAAATGGAG |
| *MaASMT2* | ATTGCTGATTTGCAGGGAAG | TCTTCGTCACTCCAGTCGTG |
| *MaASMT3* | GTTGCGTCTGTTGCAGAAAA | AAAATGCACGACGAAATCCT |
| *MaASMT4* | CCGATGAGTTTGGCTGAAAT | ATCTTTAGGCGGACCAGGAG |
| *MaASMT5* | CAGACCCATCCGTGGCTGAT | TCTCACGTCCATGGGCCTTC |
| *MaASMT6* | CCATTCCCGACACTTCATCT | CCGTCTGATGATTGATGTGC |
| *MaASMT7* | ATGGAATCAGACGAGGCAAG | GGAATTTTGAGCTCCACAGC |
| *MaASMT8* | GGAGGAGCAAAACAACAAGG | TCTCGGCCAGGGTATAACAC |
| *MaASMT9* | TAGTCAATGCGTTCCCACAC | TCCGCCGATAAAATTCAAGT |
| *MaASMT10* | GGAAATACGCTGAGGCAAAC | ACACGTCCAAACAACCATCA |
| *MaASMT11* | GTGCTGTTCAATTGGGCATA | TGCTTTGTTTTGGTGGACTG |
| *MaASMT12* | TGACTCGGGGATGATGAACT | TTTGGAGATTGTTCCGGTTC |
| *MaASMT13* | TCTTGGAGGCTGGTTTCAGT | GGCCTACTTGGTCCTGATGA |
| *MaASMT14* | CGCTCCTTCTTATCCAGGTG | TCATCGGTCCAGTCATGAAG |
| *MaASMT15* | TACGGCCTTTCTCCTGTGTC | GCCAGGTATCCACAAAGACC |
| *MaASMT16* | TGTGCTCAATTGCTCTTTGG | GAAACGCCATCTTCATTCGT |
| *MaASMT17* | GCGCGTATCATGAGATTCCT | ATCTCGACAAGTGGGTCAGG |
| *MaASMT18* | TCTCGCCGGTTAATGAGAAC | GACACATGCGCTCAGACAGT |
| *MaASMT19* | CAACAAAGCAATGGACAACG | AAACCACCACCAACATCCAC |
| *MaASMT20* | GTGGTGTTGGAAGGGAGAGA | AATTGAGGGCATAGCTGGAA |
| *MaACTIN3* | GCATGAAGATCAAGGTGGTG | CATCTGCTGGAAGGTGCTAA |

Supplementary Table 3. The primer sequences for cloning of *MaASMT*s.

| Name of gene | Forward (5' - 3') primer | Reverse (5' - 3') primer |
| --- | --- | --- |
| *MaASMT4* | ATGAAACTTGACGAGGCAAG | TCACTGTGGATATGCCTCAA |
| *MaASMT9* | ATGTCTAGCAGTGATGAG | TTAAGGGTAAACCTCAATGAC |
| *MaASMT19* | ATGGAAAACAATCCAAAC | TACTTAAAGAACTCCAT |
| *MaASMT20* | ATGGAGGGAATAGATCATCATG | TTAAACTGGATAGGCCTC |

Supplementary Table 4. Parent ions and fragments following high-resolution-mass spectrometer analyses of melatonin, tryptophan-ethyl ester and its isomers.

| Name of sample | Compound | Retention time | [M+Na]+ 255.1049 | [M+H]+  233.1290 | 216.1025 | 174.0919 | 159.0684 |
| --- | --- | --- | --- | --- | --- | --- | --- |
| melatonin standard | Mel | 5.52 | N | Y | N | Y | Y |
| tryptophan-ethylester standard | tryptophan-ethylester | 5.43 | N | Y | Y | Y | Y |
| S4-L | Mel | 5.52 | N | Y | Y | Y | Y |
| S4-L | MI-1 | 4.55 | Y | Y | Y | Y | Y |
| S36-L | MI-1 | 4.55 | Y | Y | Y | Y | Y |

N: no detected, Y: detected.

Supplemental Table 5. Information regarding the *ASMT* genes in different plant species.

| Abbreviation | Full name | GenBank accession no |
| --- | --- | --- |
| OsASMT1 | *Oryza sativa* | BAG93122.1 |
| OsASMT2 | *Oryza sativa* | BAG91369.1 |
| OsASMT3 | *Oryza sativa* | AAL34949.1 |
| AtASMT | *Arabidopsis thaliana* | At4g35160 |
| MdASMT | *Malus domestica* | AIY62760.1 |
